# An Acid–Base Proton Transfer Approach to Robust Superhydrophobic Self-Cleaning Surfaces for the Corrosion Protection of Magnesium

**DOI:** 10.3390/ma18051028

**Published:** 2025-02-26

**Authors:** Junjie Chen, Baoshan Xu, Yunhao Zhao, Ke Zhou, Ruijuan Shao, Xiaowei Xun, Fan Zhang, Dongmian Zang

**Affiliations:** School of Materials Science and Engineering, East China Jiaotong University, Nanchang 330013, China; 18333836056@163.com (J.C.); xbshan@126.com (B.X.); 17549209589@163.com (Y.Z.); zhouke0301@163.com (K.Z.); 18896651032@163.com (R.S.); xunxiaowei@ecjtu.edu.cn (X.X.); zhang_fan2003@126.com (F.Z.)

**Keywords:** Mg corrosion, functional coating, superhydrophobicity, durability, self-cleaning

## Abstract

In this study, an acid–base proton transfer method was used to fabricate functional coatings on Mg surfaces with the cooperative effect of durable superhydrophobic and exceptional self-cleaning properties, providing high-efficiency corrosion protection. In this context, aluminum chloride served as a precursor for the direct growth of aluminum hydroxide on the Mg surface. Without the addition of any solvent, the densely arranged absolute palmitic acid was strongly bonded to the grown aluminum hydroxide on the Mg substrate, which acted as an effective anti-water barrier that can impede the penetration of water, as well as the oxygen and chloridion involved.

## 1. Introduction

Metal corrosion naturally occurs, leading to economic expenses, safety hazards, environmental damage, and an accelerated depletion of natural assets. As such, nature-inspired superhydrophobic metallic surfaces have been carefully created to afford long-term protection against corrosion. These surfaces exhibit exceptional water repellency, efficiently shielding metals from detrimental moisture and corrosive conditions, thereby extending the durability and service life of metal materials [1,2,3,4]. The manufacture of such surfaces offers a promising solution for mitigating the enormous economic and structural costs associated with metal corrosion [5,6]. In this context, Mg and its alloys have found widespread applications in the aviation, automotive, and 3C fields, resulting from favorable attributes such as its lightweight nature, superior specific strength, and outstanding thermal conductivity [7,8,9,10,11]. Nonetheless, their inherent high chemical reactivity and low electrode potential lead to a vulnerability to corrosion, thereby constraining their practical utilization. In this instance, to improve the resistance of Mg alloys to corrosion, superhydrophobic surfaces have emerged as a widely adopted technology, applied across a diverse range of Mg alloy surfaces [12,13,14,15].

As is well known, solid–liquid contact reduction governs hydrophobicity enhancement. However, a minimal solid–liquid interface, causing structural vulnerability and limited wear endurance, poses a pivotal obstacle to the broad implementation of conventional superhydrophobic surfaces. Consequently, considerable endeavors have been undertaken to fortify these surfaces against degradation under adverse environments, aiming to address this issue. In this regard, the high bonding strength of a substrate is crucial for improving the mechanochemical stability. Chemical bonding can improve the adhesion strength of film–substrate interfaces, guaranteeing its robustness and engineering application. It is widely acknowledged that acids can form stable chemical bonds with metal oxides and hydroxides. He and his team created a durable superhydrophobic coating by bonding myristic acid to the anodic aluminum oxide layer on aluminum [16]. Similarly, Duan and colleagues reported that a ZnAl–LDH–laurate film exhibited robust superhydrophobic properties, effectively repelling aggressive species from an Al surface [17]. In this context, Yu et al. developed Mg alloys featuring durable superhydrophobic surfaces by bonding stearic acid (SA) to the rough structure of Fe(OH)_3_ films [18]. Additionally, Cu foam coated with a CuS@Cu(OH)_2_ nanocomposite and modified with palmitic acid (PA) exhibited superhydrophobic properties, high robustness, corrosion resistance, and an effective separation of oily wastewater [19]. In particular, Wang et al. applied a SA–Al(OH)_3_ film onto lignocellulose composites to impart superhydrophobic properties assisted by polydimethysiloxane (PDMS) [20]. In this instance, SA and PDMS are both classified as hydrophobic materials characterized by low surface energy [18,21,22].

Here, we demonstrated that the chemical bonding of PA with aluminum hydroxide which is grown directly on a Mg surface results in the formation of a durable, hierarchical micro/nanostructured superhydrophobic film without the use of PDMS. In this context, aluminum hydroxide (AH) coatings were grown onto Mg substrates using the in situ water bath solution immersion technique, utilizing aluminum chloride as a precursor. Subsequently, these AH coatings were functionalized with PA to produce superhydrophobic Mg (SM) surfaces. The resultant SM surfaces demonstrated remarkable stability across a range of temperatures, including high temperatures and room temperature, as well as exposure to acidic, alkaline, and saline environments. Furthermore, they exhibited exceptional mechanical durability, as evidenced by water flow impact, grit impact, and sandpaper friction tests. Notably, SM surfaces possessed exceptional self-cleaning capabilities and significantly mitigated the corrosion of the magnesium substrate. It is believed that our straightforward fabrication method can be easily adapted for the creation of durable superhydrophobic surfaces on magnesium alloys and magnesium matrix composites, which may hold considerable potential for a wide range of engineering applications.

## 2. Materials and Methods

### 2.1. General Materials

The magnesium plate, with a purity of 99.99%, originated from Baowu Magnesium Technology Co., LTD, Nanjing, China. The chemicals utilized comprised PA (98%), NaOH (96%), HCl (37%), and C_2_H_5_OH (99.7%), all sourced from J&K Scientific in Beijing, China. And aluminum chloride hexahydrate (AlCl_3_·6H_2_O, 95%) and acetone (CH_3_COCH_3_, ≥99.5%) were acquired from Xilong Science Ltd., located in Shantou, China. Additionally, these chemicals were used without any further purification. Throughout all experimental procedures, deionized water from the MilliQ Academic A10 Water Purification System was chosen as the solvent.

### 2.2. Instruments

The microstructure images of the samples were captured through the use of Scanning Electron Microscopy (SEM, Hitachi SU8010, Tokyo, Japan) and Atomic Force Microscopy (AFM, Cypher ES AFM, Santa Barbara, CA, USA). Prior to SEM imaging, the specimens underwent gold sputtering coating. Additionally, AFM imaging was carried out under standard environmental conditions. The elemental composition and chemical state were examined utilizing energy-dispersive X-ray spectroscopy (EDS) integrated within the SEM instrument and the X-ray Photoelectron Spectroscopy (XPS). The XPS analysis was performed on a Thermo Scientific Escalab 250Xi system, Waltham, MA, USA, employing a 200 W monochromated Al Kα radiation source with a 500 μm X-ray spot size. The analysis chamber was maintained at a base pressure of approximately 3 × 10^−10^ mbar. The sessile drop method was employed for water contact angle (CA) measurements, utilizing a Dataphysics OCA15Pro system (Dataphysics, Filderstadt, Germany) at an ambient temperature. To evaluate the static CA, a precisely measured 5 μL sessile droplet was deposited onto the sample surface. Once the droplet stabilized, side-view images were taken. To ensure accuracy, static CA was averaged by five separate measurements at distinct locations on the same specimen. Similarly, the roll-off angles were evaluated under the same environmental conditions as those used for the static CA testing. The electrochemical assessment was performed in an aqueous solution containing 3.5 wt% NaCl at an ambient temperature, utilizing the CHI 760 D analyzer (CH Instruments, Shanghai, China) with a standard 3-electrode setup. The magnesium was used as the working electrode with a 1 cm^2^ exposed surface, a Pt plate served as the counter electrode, and a saturated calomel electrode (SCE) acted as the reference electrode. After allowing the electrochemical system to stabilize for 0.5 h, the electrochemical impedance spectroscopy (EIS) measurements were conducted across a frequency range of 100 kHz to 10 mHz, with the signal amplitude perturbation set at 5 mV. The Tafel polarization test was performed, and the scan rate was 1 mV/s. A Tafel plot was obtained by carrying out partial potentiodynamic polarization in the potential range ± 500 mV from open-circuit potential. Tafel curves were used to extrapolate the corrosion potential (E_corr_) and corrosion current density (I_corr_) values.

### 2.3. Treatment of Magnesium

The magnesium sample was cut to a size of 2 cm × 4 cm × 0.2 cm, followed by mechanical polishing using sandpapers of different grit sizes. Each sample underwent a rigorous cleaning process involving immersion in a bath of ethanol, deionized water, and acetone for five minutes at room temperature, with continuous ultrasonic treatment to eliminate impurities. Following nitrogen drying, the magnesium samples were dipped in 0.1 mol/L aluminum chloride water solution maintained in a water bath for a period of three hours at 50 °C to facilitate the growth of an AH film on its surface. Subsequently, the AH-coated Mg (AHM) underwent modification using pure PA at a temperature of 85 °C for 1.5 h, with the process being carried out without the incorporation of any solvent, significantly reducing both preparation costs and resource consumption. After modification, the resulting magnesium plates were swiftly rinsed with hot ethanol and placed in an oven to dry at 80 °C for thirty minutes.

### 2.4. Self-Cleaning Test

In order to characterize the magnesium surface’s self-cleaning capability, yellow sand particles of varying sizes were employed as contaminants. The magnesium surfaces were inclined at an angle of 5 degrees. A plastic dropper was utilized to dispense water droplets at a consistent rate, positioning them close to the magnesium surface. These sand particles were randomly placed onto the magnesium surface, followed by dripping the water droplets directly from the plastic dropper onto the surface, simulating the self-cleaning process [21].

### 2.5. Water Flow Shear Test

The SM was immobilized within PDMS and subsequently exposed to water, with the water flow directed at a 90° angle to the magnesium surface. The flow rate of the water could be adjusted using a flow control valve. During this experiment, we employed three varying water flow rates: 5 cm/s, 10 cm/s, and 15 cm/s, to investigate the ability of the SM surface to withstand the force of water flow shear. The water CA was documented at intervals of 0.5 h for a period of 2 h.

### 2.6. Grit Impact Test

Fine sand particles, ranging in size from 100 to 250 μm, were dropped and continuously impinged the SM at a 45-degree angle. The drop height of the sand particles was 300 mm. In this context, the CA of water was documented at intervals of 5 min, spanning a total duration of 25 min.

### 2.7. Sandpaper Friction Test

A magnesium sample loaded with a 100 g weight was moved horizontally across sandpaper (1000 grit) by applying an external force, with the water CAs being recorded at every 20 cm interval [6].

### 2.8. Chemical Stability Test

The magnesium surface’s apparent water CA was evaluated across a broad spectrum of pH values. By adjusting the pH of the water droplets using HCl and NaOH, the magnesium was subjected to a range of acidic, alkaline, and saline conditions, including the entire pH range from 1 to 14 [6,18].

### 2.9. Durability Assessment

The magnesium samples were positioned in a controlled lab environment for exposure to atmospheric conditions at ambient temperature for a period of 16 months. During this time, water CA measurements were recorded at regular intervals of every 4 months [18].

### 2.10. Heat Stability Test

The magnesium specimens were positioned inside an oven kept at a temperature of 160 degrees Celsius for a duration of 36 h, during which the water CAs were assessed both prior to and subsequent to the heating process, with measurements conducted every 6 h.

## 3. Results and Discussion

### 3.1. Fabrication of SM Surfaces

**Figure 1:**
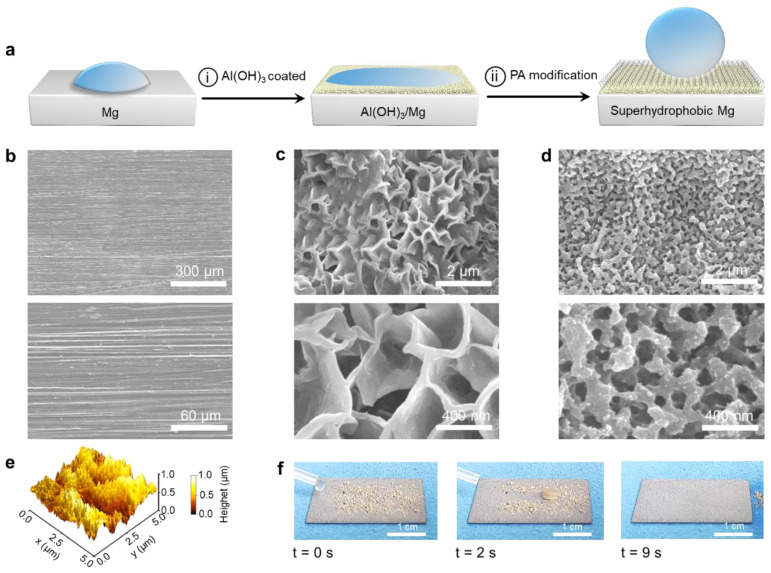
Schematic design for the SM manufacturing along with the pertinent characterizations. Schematic fabrication of the SM surface (**a**). The SEM morphologies of the surface of original Mg (**b**), AHM (**c**), and the resultant SM (**d**). AFM image of the SM surface, offering a detailed view of its surface texture (**e**). Self-cleaning capability of the SM surface (**f**).

Figure 1a illustrates the process of creating SM surfaces. Initially, an AH coating was grown on the Mg surface, utilizing aluminum chloride as the precursor. As such, the following simplified chemical reaction was involved: 3Mg + 2AlCl_3_ + 6H_2_O = 3MgCl_2_ + 2Al(OH)_3_ + 3H_2_. During this process, the hydrolysis reaction of the involved Al^3+^ brought about the formation of an AHM. Subsequently, the Mg surface underwent chemical bonding through the mediation of AH with pure PA, resulting in the formation of an SM surface.

As depicted in Figure 1b, the SEM images of Mg exhibit a relatively smooth surface, characterized by an abundance of machining lines from the grinding and polishing process. After the growth of AH on the Mg surface, the AHM was covered with a sea of petals that were irregular polygons, closely arranged and overlapped with each other. The petal thickness measured approximately 50 nm, while the average diameter of the cavities between the petals was 350 nanometers. The development of these cavities might be linked to the hydrogen evolution process occurring during the formation of AH. In this regard, the nanoscale cavities could offer anchoring sites for subsequent PA modification, thereby improving the adhesive strength at the interface (Figure 1c). When the porous matrix was chemically bonded with pure PA, a more compact surface was obtained in comparison to that of the AHM. In this context, the diameter of the cavity was about 260 nanometers less than that of AHM, and the petal thickness increased to 140 nanometers larger than that of AHM (Figure 1d). Figure 1e presents a three-dimensional AFM image showcasing the superhydrophobic surface of magnesium. Within the scanned 5 μm × 5 μm area, the Mg surface featured numerous cavities and islands that aligned with the morphological and structural attributes observed in SEM images. The surface roughness, measured as the root mean square (R_a_), was 165.7 nm. Figure 1f displays the self-cleaning property of the SM surface. At the beginning (t = 0 s), the yellow sand was arbitrarily distributed across the surface of the SM. As time moved forward to t = 2 s, a distinct self-cleaning track emerged on the SM surface due to the motion of the water droplet. Consequently, within a span of 9 s, the moving water droplet eliminated every last trace of yellow sand from the surface, highlighting the impressive self-cleaning property of the SM.

**Figure 2:**
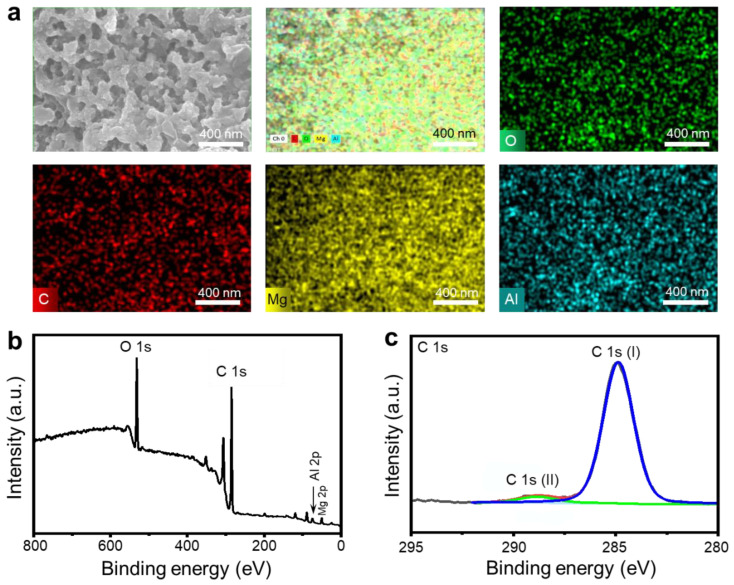
The compositional analysis of the SM surface by EDS and XPS. The corresponding EDS maps (**a**) and XPS wide scan spectrum (**b**) of the SM. High-resolution XPS spectrum of the SM in the C 1s spectral region (**c**).

The surface chemistry of the SM was analyzed through the utilization of EDS and XPS techniques. In this context, EDS mappings reveal the existence of magnesium, aluminum, oxygen, and carbon elements on the SM (Figure 2a). Additionally, XPS analysis confirmed the presence of Mg 2p, Al 2p, O 1s, and C 1s peaks in its survey spectra, as depicted in Figure 2b. The results aligned well with those from the EDS test. In this case, the sharp C 1s peak exhibited a symmetrical spectrum, accompanied by a less intense shoulder peak in the region of higher binding energy (BE), as shown in Figure 2c. Peak fitting analysis revealed two distinct constituents: one located at a BE of 284.8 eV designated as C 1s (I), and another at 288.8 eV designated as C 1s (II). The primary component, C 1s (I), corresponded to aliphatic carbon, whereas C 1s (II) was associated with carboxyl groups bonded to the AHM surface [23,24,25]. This coordination facilitated the creation of the SM mediated by robust chemical bonds between the PA and the AHM, thereby improving the adhesion between the film and the substrate.

**Figure 3:**
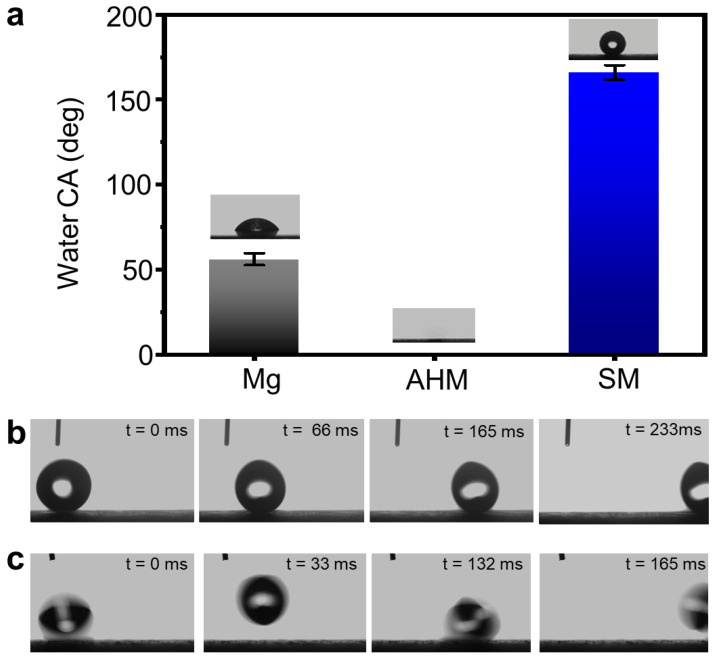
Wettability assessment of different magnesium specimens. Measurements of the water CA on the bare Mg surface, AHM surface, and SM surface (**a**). Specific snapshots captured during the experiments illustrate an 8.0 μL water droplet sliding off the SM surface in 233 ms; the SM was tilted by 5 degrees (**b**), and within 165 milliseconds, a 6.0-microliter water droplet bounded off the SM surface, while the SM remained horizontal (**c**).

Figure 3a shows that the static CA on the surface of the pristine magnesium was observed to be 56.0 ± 3.5°, whereas it decreased significantly to 0° on the AHM surface. In this context, a sea of hydroxyl groups on the AHM surface and its rough structure reduced the CA. Subsequently, the superhydrophilic AHM with roughened surface was hydrophobized by PA, leading to the formation of SM. The SM surface exhibited a static water CA of 159.0 ± 2.3°. As illustrated in Figure 3b, the water droplets could roll off from the SM surface within 233 milliseconds when the surface was inclined by 5 degrees. Additionally, upon impacting the SM sample, the water droplets displayed a bouncing behavior, swiftly detaching from the surface within just 165 milliseconds (Figure 3c). These findings suggest that the CA hysteresis was ultralow and the surface exhibited non-wetting behavior in parallel with Cassie’s model [26,27].

### 3.2. Mechanochemical Durability of SM Surfaces

**Figure 4:**
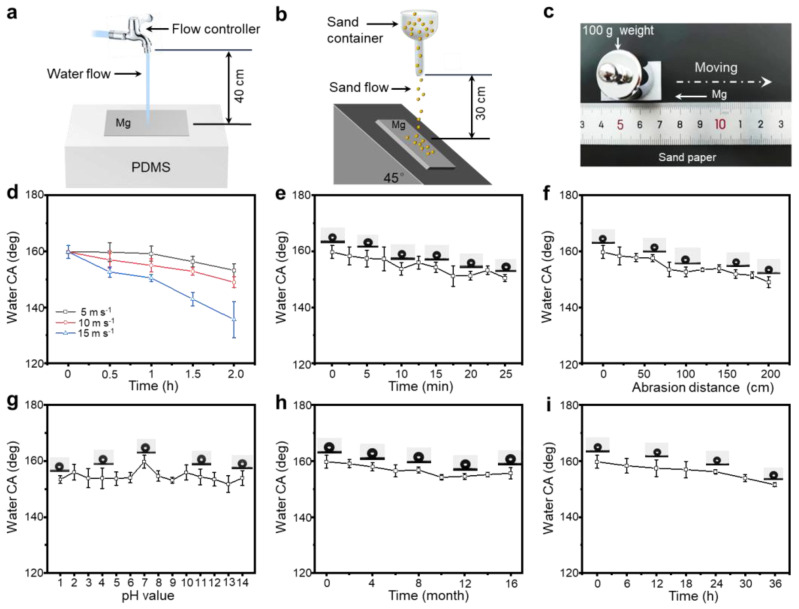
The mechanochemical durability characteristic of the SM surface. Illustrations depicting the experimental setups include: a schematic for the water impact test (**a**) and the grit impact test (**b**), as well as an illustrated representation of the sandpaper friction testing process (**c**). The relationship between water flow shearing time and the static water CA (**d**), the duration of grit impact and the static CA of water (**e**), as well as the dependence of static water CA on the sandpaper abrasion distance (**f**). The effects of pH levels (**g**), exposure duration to air (**h**), and resistance time at high temperatures (**i**) on the static water CA.

It has been well established that superhydrophobic surfaces necessitate mechanochemical robustness to ensure outdoor applications [28,29,30,31]. In this context, the ability of a superhydrophobic self-cleaning surface to withstand water flow shear is essential for its practical applications. Figure 4a shows the performed water flow shear test on the SM. In this case, the SM’s superhydrophobic property was maintained for 2.0 h when the water flowed at speeds of 5 cm s^−1^ and 10 cm s^−1^. However, as the water flow rate increased to 15 cm s^−1^, the duration of superhydrophobicity decreased to 1.0 h (Figure 4d). As illustrated in Figure 4b, the sand impact test was carried out. After enduring grit impact for 25 min, it is evident from Figure 4e that the water CA on the surface remained above 150°. As such, the persistent grit impact had no detrimental effect on the superhydrophobic properties. Furthermore, the SM’s mechanical robustness was assessed through sandpaper friction experiments, as depicted in Figure 4c. Notably, during the loaded movement of the SM over a substantial distance of 200 cm, the CA of water on the SM surface consistently exceeded 150° (Figure 4f).

In addition, to assess the chemical stability of SM, the pH level of the water droplet was adjusted by HCl and NaOH. These droplets, spanning a pH range of 1 to 14, served as measuring tools for determining the water CA. As illustrated in Figure 4g, the water CA remained consistently greater than 150° across all pH values tested (1–14) on the SM, demonstrating its remarkable chemical stability exposure to highly acidic, alkaline, or saline conditions. Figure 4h illustrates that the water CA on the SM surface maintained a high value of 155.6 ± 2.0° even after 16 months in air, indicating the superhydrophobic surface’s sustained durability in ambient conditions. It is noteworthy that the SM surface exhibited exceptional high-temperature resistance, enduring temperatures up to 160 °C. Over this duration, the CA underwent minimal change, with a value of 151.5 ± 0.7° observed after 36 h, as illustrated in Figure 4i.

These results offer compelling evidence of the exceptional mechanochemical properties exhibited by SM. This is because of the direct growth of AH on the magnesium surface, where the structure of AH film was textured, as well as the proton transfer between PA and AH facilitated the formation of a robust chemical bond.

### 3.3. Resistance to the Corrosion of SM Surfaces

**Figure 5:**
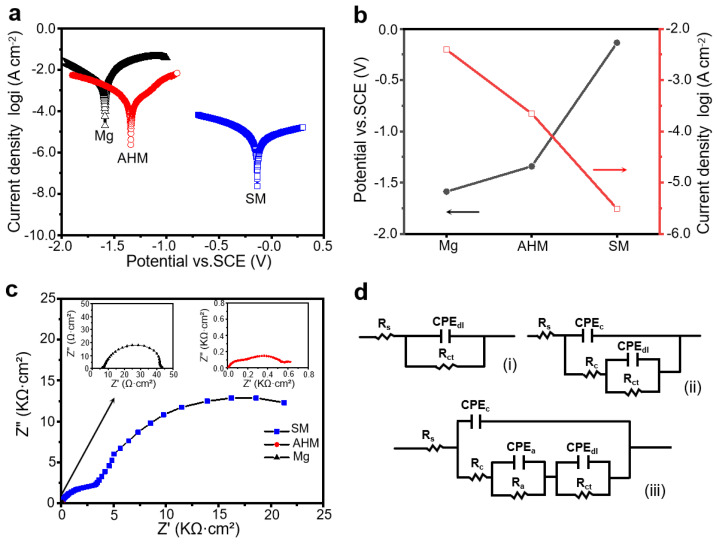
The electrochemical behaviors of the untreated Mg, AHM, and SM. Tafel polarization curves of the as-received magnesium, AHM, and SM (**a**). A comparison panel showing the E_corr_ and Icorr of the pristine Mg, AHM, and SM (**b**). Nyquist plots of the Mg, AHM, and SM (**c**). Equivalent electrical circuits tailored to fit the EIS spectra of different samples, specifically for Mg (i), AHM (ii), and SM (iii) (**d**).

To evaluate the corrosion resistance of SM, Tafel curve tests were performed on different Mg specimens. The Tafel curves and their respective fitted values are presented in Figure 5a,b. The pristine Mg exhibited a E_corr_ of −1.588 V (SCE) and a I_corr_ of 3.914 × 10^−3^ A cm^−2^. In this context, when exposed to a highly corrosive environment containing chloride ions, the Mg specimen underwent pitting corrosion accompanied by hydrogen evolution [12]. After applying an AH film, the E_corr_ of the AHM shifted positively to −1.342 V (SCE), with the I_corr_ decreasing to 2.217 × 10^−4^ A cm^−2^. Following PA modification, the SM surface exhibited superhydrophobic properties, resulting in a E_corr_ of −0.135 V (SCE), which was notably elevated compared to the original Mg. The I_corr_ further decreased to 3.907 × 10^−6^ A cm^−2^, representing a three-order-of-magnitude reduction compared to bare Mg, thereby demonstrating exceptional corrosion resistance. In this context, the inhibition efficiency (IE) can be determined using the following formula in terms of I_corr_: IE = (1 − I_corr_ (film)/I_corr_ (bare)) × 100%, where I_corr_ (film) denotes the I_corr_ of film-deposited magnesium, and I_corr_ (bare) represents the I_corr_ of the bare magnesium. The estimated IE values for AHM and SM were 94.336% and 99.900%, respectively. These findings highlight that the enhanced protective qualities of SM surfaces substantially altered their corrosion behavior, offering considerable potential for inhibiting Mg corrosion.

To gain deeper insights into the corrosion characteristics of various Mg electrodes, EIS measurements were performed in an aqueous solution containing 3.5 wt% sodium chloride. Figure 5c displays the EIS plots for three distinct samples: bare Mg, AHM, and SM. The Nyquist plot obtained from the uncoated Mg sample exhibited a relatively low real impedance magnitude, featuring a single semicircle, which suggests significant H_2_ evolution in a NaCl solution. In contrast, the plots for the AHM and SM surfaces displayed more intricate double semicircles. The semicircle appearing in the higher frequency range corresponded to a fast charge transfer, whereas the one in the lower frequency range indicated a slower process [32,33]. The shape transformation as well as the notable elevation of the real impedance magnitude suggests that SM exhibited minimal H_2_ evolution when served in a corrosive environment containing chloride ions, thereby demonstrating exceptional corrosion resistance.

As depicted in Figure 5c, the capacitive loops exhibited deviations from perfect semicircular shapes due to the dispersing phenomenon. The presence of this dispersing effect in impedance behavior indicated that the electrical double layer (DL) does not behave as a perfect capacitor [34]. As such, a constant phase element (CPE) is often used in place of the capacitor in the equivalent circuit (EC), allowing for a finer depiction of the electrical DL’s impedance characteristic. Specifically, the admittance (Y) and impedance (Z) can be mathematically expressed as follows: Y_CPE_ = Y_0_ (jω)^n^ and Z_CPE_ = 1/Y_CPE_, respectively, where Y_0_ represents the CPE’s magnitude, ω signifies the angular frequency, j^2^ = −1 indicates the imaginary number, as well as n denotes the exponential term of CPE. In this context, the CPE value was contingent upon ω. Figure 5d (I) illustrates an EC model that captures the electrochemical property of the uncoated Mg surface. In this EC, R_ct_ represents the resistance to charge transfer, CPE_dl_ denotes the CPE associated with the electrical DL, and the resistance of the solution is abbreviated as R_s_. Figure 5d (II) depicts an EC model that characterizes the electrochemical property associated with the AHM surface which exhibits superhydrophilic property. In this instance, the AH coating resistance is abbreviated as R_c_, and the CPE of the AH coating is abbreviated as CPE_c_. When the Mg was protected by a superhydrophobic coating, the corresponding EC model of the SM is depicted in Figure 5d (III). The model incorporates R_a_ and CPE_a_, where R_a_ signifies the air resistance, and CPE_a_ represents the CPE associated with the air, which is resultant from the captured plenty of air within the nanocavities of the SM.

As illustrated in Figure 5c and Table 1, the R_ct_ values of Mg samples exhibited a notable increase, changing from Mg (3.761 × 10 Ω cm^2^) to AHM (5.353 × 10^2^ Ω cm^2^), and ultimately to SM (2.435 × 10^4^ Ω cm^2^). As such, the greater the R_ct_, the lower the I_corr_. Consequently, the IE can be calculated using the formula derived from the R_ct_: IE = (1 − R_ct_ (bare)/R_ct_ (film)) × 100%, where the R_ct_ (film) denotes the R_ct_ of the film deposited magnesium, and the R_ct_ (bare) represents the bare magnesium’s R_ct_. The calculated IEs for AHM and SM were 92.974% and 99.846%, respectively. These findings align with the IEs from the Tafel curves, indicating that our superhydrophobic coating exhibited significant effectiveness in inhibiting Mg corrosion.

**Figure 6:**
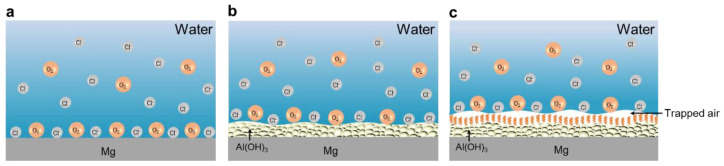
Interfacial model elucidating the corrosion resistance mechanism for the untreated Mg (**a**), AHM (**b**), and SM (**c**) surfaces in a corrosive environment containing chloridion.

Figure 6 illustrates the basic mechanism of corrosion resistance exhibited by the SM in an aggressive environment containing chloride ions. In this context, due to the porosity and instability of the native MgO layer, the Mg substrate was susceptible to direct contact with corrosive media. Consequently, aggressive Cl^−^ and O_2_ can readily “invade” the Mg substrate, causing severe corrosion for the magnesium (Figure 6a). Once the substrate is coated with the AH layer, it acts as a protective barrier, significantly hindering the penetration of water, corrosive Cl^−^ and O_2_ to the Mg substrate (Figure 6b). As shown in Figure 6c, the SM efficiently trapped air within the valleys situated between the peaks of the textured structure. As such, a curved interface, referred to as a capillary interface, was formed between the water and the entrapped air [35]. In this instance, the air-filled valleys within the roughened structure of the SM served as an efficient additional barrier, effectively hindering the penetration of water, aggressive Cl^−^ and O_2_, thereby imparting exceptional resistance of corrosion for the magnesium substrate.

Concurrently, as shown in Table 2, a comparative assessment was conducted between the properties of our SM and those exhibited by the superhydrophobic magnesium or magnesium alloy materials reported in existing literatures involved in terms of water CA, mechanical durability, and corrosion resistant properties. The findings demonstrate that our SM surpassed the other superhydrophobic Mg or Mg alloys with respect to its comprehensive performance [36,37,38,39,40,41].

## 4. Conclusions

To summarize, we demonstrated robust superhydrophobic self-cleaning surfaces for the corrosion protection of magnesium, which were accomplished by functionalizing the grown AH with pure PA based on magnesium using an acid–base proton transfer method. As such, the resultant surface exhibited exceptional mechanical durability, proven by its ability to resist water impact, grit impact, and sandpaper abrasion, as well as its robust chemical stability. In addition, this robustness persisted under both ambient conditions and at elevated temperatures. Notably, the durable superhydrophobic coating served as an efficient shield, effectively mitigating magnesium corrosion. Given its straightforward synthesis, cost-effectiveness, and manageable process, our preparation approach offers significant potential for enhancing magnesium-based materials in corrosion resistance, self-cleaning capability, and super-wetting characteristic, enabling wider industrial applications.

## Figures and Tables

**Table 1:** Fitted EIS parameters for pristine Mg, AHM, and SM.

Sample	R_s_/(Ω·cm^2^)	CPE_dl_	n	R_ct_/(Ω·cm^2^)	CPE_c_	n	R_c_/(Ω·cm^2^)	CPE_a_	n	R_a_/(Ω·cm^2^)
		Y_0_/(Ω^−1^ cm^−2^ s^n^)			Y_0_/(Ω^−1^·cm^−2^ s^n^)			Y_0_/(Ω^−1^·cm^−2^ s^n^)		
Mg	6.839	5.193 × 10^−5^	0.9161	3.761 × 10^1^	—	—	—	—	—	—
AHM	8.230	5.975 × 10^−4^	0.4926	5.353 × 10^2^	2.671 × 10^−5^	0.8126	1.901 × 10^2^	—	—	—
SM	1.461	3.904 × 10^−5^	0.9713	2.435 × 10^4^	2.008 × 10^−6^	0.9999	1.287 × 10^3^	5.388 × 10^−6^	0.9945	2.502 × 10^3^

**Table 2:** Comparative analysis of the properties of different superhydrophobic Mg or Mg alloys.

Substrate	Surface Coating	Water CA	Mechanical Durability	I_corr_ Decreased by Orders of Magnitude	Application	Ref.
Mg	LDH/Sodium oleate	151.2 ± 2.4°	The length of abrasion = 50 cm (2.45 kPa, 1000 grit sandpaper)	2	Anti-corrosion Biomedicine	[36]
AZ31B	Crystalline solid myristic	156.2 ± 2°	/	2	Anti-corrosion	[37]
Mg	LDH/Myristic acid	152.2°	/	1	Anti-corrosion	[38]
Mg-Li-Ca alloy	MAO/Stearic acid	155.5°	/	3	Anti-corrosion	[39]
AZ31B	Ni-phosphorus/Ni/PFDTMS	153.0 ± 4.6°	/	2	Anti-corrosion	[40]
AZ31B	Zn-Fe/Myristic acid	153.0°	The length of abrasion = 1 m (2.45 Pa, 2000 grit sandpaper)	1	Anti-corrosion Self-cleaning	[41]
Mg	Al(OH)_3_/Palmitic acid	159.0 ± 2.3°	(1) The length of abrasion = 200 cm (100 g weight, 1000 grit sandpaper). (2) The time of water flow shear = 2.0 h (speed = 10 cm/s). (3) The time of grit impact = 25 min (drop height = 30 cm).	3	Anti-corrosion Self-cleaning	This work

## Data Availability

The original contributions presented in the study are included in the article, and further inquiries can be directed to the corresponding author.
